# Elevated methylmalonic acid, but not vitamin B12, predicts all-cause mortality in hyperlipidemic adults: a prospective cohort study

**DOI:** 10.3389/fnut.2026.1742540

**Published:** 2026-01-15

**Authors:** Qingtao Gong, Baiqiang Wang, Leiyang Li, Gongshuang Zhao, Chengzhi Li, Lianyue Ma, Hong Yang, Xiaojuan Zhang, Guipeng An, Chenghu Guo

**Affiliations:** 1 Public Health Clinical Center Affiliated to Shandong University, Shandong University, Jinan, China; 2 State Key Laboratory for Innovation and Transformation of Luobing Theory, Key Laboratory of Cardiovascular Remodeling and Function Research of MOE, NHC, CAMS and Shandong Province, Department of Cardiology, Qilu Hospital of Shandong University, Jinan, China; 3 Jinan Vocational College of Nursing, Jinan, China

**Keywords:** all-cause mortality, hyperlipidemia, methylmalonic acid, prognosis, vitamin B12

## Abstract

**Objectives:**

Despite lipid-lowering therapy, patients with hyperlipidemia retain significant residual risk. This study investigated the independent and combined associations of serum methylmalonic acid (MMA), vitamin B12, and functional vitamin B12 status with all-cause mortality in this population.

**Methods:**

We analyzed data from hyperlipidemic participants in the National Health and Nutrition Examination Survey (NHANES) from 2011 to 2014. Weighted Cox proportional hazards models were employed to evaluate the relationships between serum MMA, vitamin B12, functional vitamin B12 deficiency (defined as elevated MMA despite normal/high B12 levels), and all-cause mortality. Nonlinear associations were examined using restricted cubic splines.

**Results:**

During a median follow-up of 6.8 years, 626 deaths occurred. After full adjustment, each unit increase in ln-transformed MMA was associated with a 55% higher mortality risk (adjusted HR = 1.55, 95% CI: 1.30–1.84, *p* < 0.001), while serum vitamin B12 alone showed no significant association. Notably, participants with functional vitamin B12 deficiency (vitamin B12 > 400 pg./mL and MMA > 250 nmol/L) exhibited the highest risk (adjusted HR = 2.40, 95% CI: 1.70–3.41, *p* < 0.001).

**Conclusion:**

Elevated serum MMA and functional vitamin B12 deficiency are significantly associated with increased all-cause mortality in hyperlipidemia, whereas serum vitamin B12 level alone lacks independent prognostic value. MMA may serve as a novel biomarker for mortality risk in hyperlipidemia population, highlighting the clinical importance of assessing functional vitamin B12 status.

## Introduction

Hyperlipidemia, a common metabolic disorder characterized by elevated levels of total cholesterol, triglycerides, and low-density lipoprotein along with reduced high-density lipoprotein, represents a major public health challenge (1). In the United States, it affects over 53% of adults and 10.7% of adolescents—a prevalence expected to rise despite improving living standards (2, 3). As a well-established driver of atherosclerotic cardiovascular disease, hyperlipidemia significantly increases the risk of stroke, coronary artery disease, and overall mortality (4). A thorough understanding of its risk factors is therefore essential for developing effective preventive and therapeutic strategies.

Vitamin B12 serves as a crucial cofactor in the metabolism of methylmalonic acid (MMA) and homocysteine (Hcy). Its deficiency leads to accumulation of these metabolites, which is associated with permanent neurological impairment, anemia, bone loss, and elevated risk of cerebrovascular and cardiovascular events (5). Among biomarkers of vitamin B12 deficiency, serum MMA is recognized as the most sensitive and specific, outperforming both serum vitamin B12 and Hcy (6). Beyond its role in vitamin status assessment, MMA has emerged as an indicator of mitochondrial dysfunction and oxidative stress (7, 8). Clinically, elevated MMA levels are linked to poorer outcomes in coronary heart disease, acute myocardial infarction, diabetes, and metabolic fatty liver disease (9–11). Critically, emerging prospective evidence solidifies MMA as an independent predictor of mortality in patients with established cardiovascular disease. A key study specifically investigating patients with pre-existing CHD demonstrated that higher serum MMA levels were strongly associated with increased risks of both all-cause and cardiovascular mortality, whereas serum vitamin B12 concentration, dietary B12 intake, and B12 supplement use showed no significant association. Notably, this mortality risk linked to elevated MMA was particularly pronounced among participants with sufficient serum B12 levels, suggesting that MMA accumulation may indicate a functional B12 deficiency or altered metabolic state relevant to disease progression (11). This evidence directly clarifies the link between MMA and hard cardiovascular outcomes, positioning it as a biomarker of metabolic disturbance that predicts mortality risk independent of conventional B12 status in CHD patients.

Elevated MMA in the context of normal or high serum vitamin B12 may reflect impaired cellular uptake or utilization of the vitamin—a condition termed “functional B12 deficiency” or “reduced B12 sensitivity.” This phenotype has been associated with accelerated biological aging and adverse long-term prognosis (12, 13). Interestingly, animal studies suggest that MMA may modulate cholesterol metabolism, reducing hepatic and plasma cholesterol levels in mice (14). However, the clinical relevance of MMA in human hyperlipidemia remains poorly understood.

To our knowledge, no previous study has examined the relationship between vitamin B12, MMA, and mortality specifically in a hyperlipidemic population. To address this gap, we conducted a large population-based cohort study aiming to elucidate whether MMA and functional vitamin B12 status can serve as prognostic biomarkers and improve risk stratification in patients with hyperlipidemia.

## Methods

### Study population

**Figure 1:**
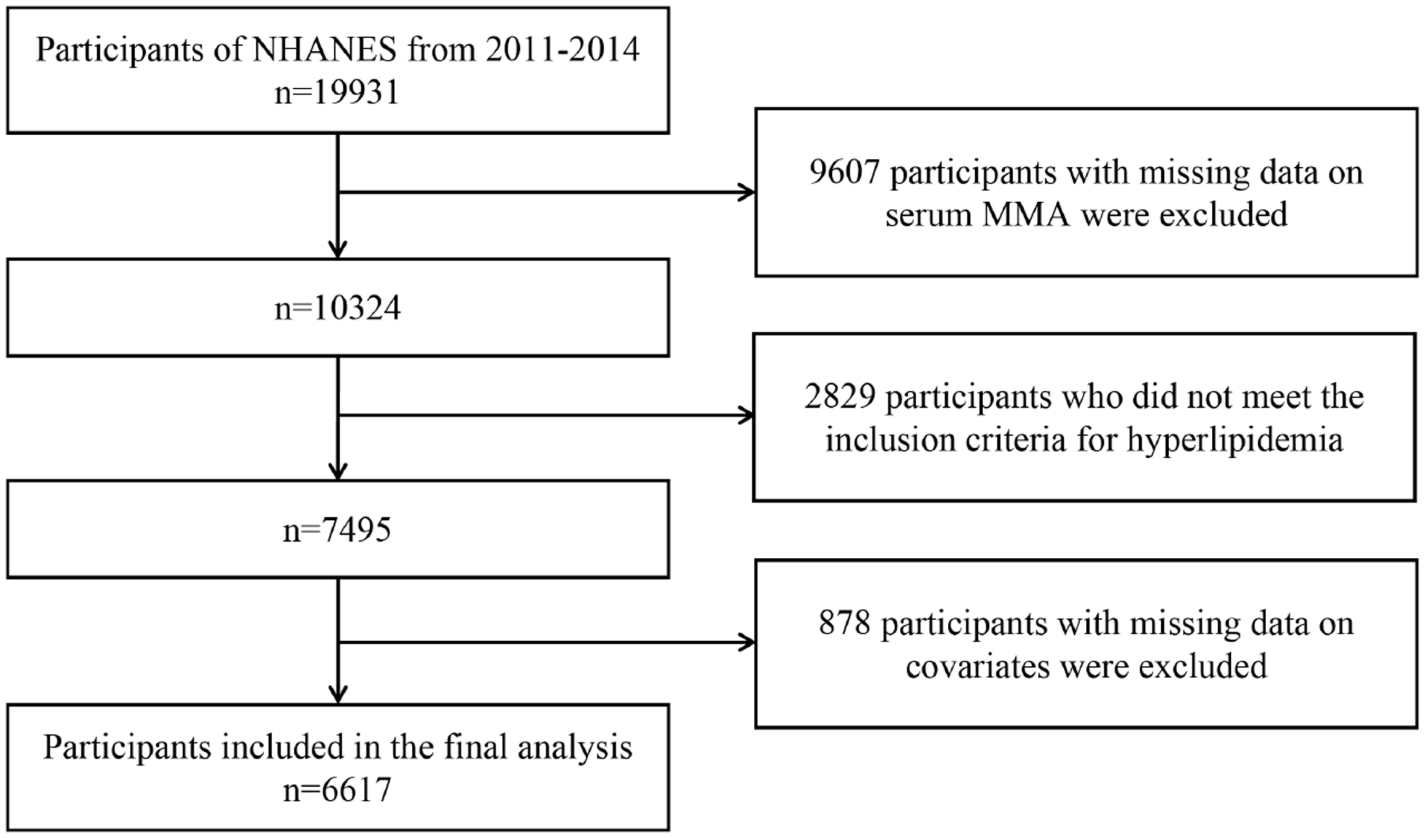
Flowchart of patient study enrollment.

This study used data from the 2011–2014 cycles of the National Health and Nutrition Examination Survey (NHANES). NHANES is conducted by the National Center for Health Statistics (NCHS) under the US Centers for Disease Control and Prevention, and is an ongoing nationwide survey designed to monitor the health and nutritional status of the US population. Participants were recruited using a complex, multistage sampling design to ensure national representativeness. All participants provided written informed consent before participation, and the study was approved by the NCHS Institutional Review Board. Data from these two cycles included 19,931 participants. Among them, 9,607 participants with missing serum MMA data were excluded. 2,829 individuals who did not meet the diagnostic criteria for hyperlipidemia were excluded. Participants with missing data on key covariates, including MMA (*n* = 731), vitamin B12 (*n* = 7), educational level (*n* = 14), smoking status (*n* = 5), stroke history (*n* = 5), heart failure (*n* = 14), body mass index (BMI, *n* = 18), alcohol consumption (*n* = 54), specific biochemical tests (*n* = 7), and glycated hemoglobin (*n* = 9), were excluded, along with 14 participants lost to follow-up, to establish the final analytical sample. After these exclusions, 6,617 individuals with hyperlipidemia were included in the final analysis. The selection process is illustrated in Figure 1. Follow-up duration (in months) was calculated from the NHANES survey completion date to either death or the end of follow-up (December 31, 2019), whichever came first.

### Assessment of serum vitamin B12 and MMA levels

Serum samples were collected by trained phlebotomists at the Mobile Examination Center (MEC), a specialized facility for health assessments, and then sent to the central laboratory for analysis. Serum vitamin B12 levels were measured using an electrochemiluminescence immunoassay (Elecsys E170), and serum MMA levels were determined using liquid chromatography-mass spectrometry (15).

### Definition of functional vitamin B12 deficiency

Functional vitamin B12 deficiency was defined as a serum MMA level > 250 nmol/L and a vitamin B12 level > 400 pg./mL (14, 16). According to these criteria, participants were categorized into four groups based on cutoff values of serum MMA and vitamin B12: B12_low_MMA_low_ (vitamin B12 < 400 pg./mL, MMA < 250 nmol/L), B12_high_MMA_low_ (vitamin B12 > 400 pg./mL, MMA < 250 nmol/L), B12_low_MMA_high_ (vitamin B12 < 400 pg./mL, MMA > 250 nmol/L), and B12_high_MMA_high_ (vitamin B12 > 400 pg./mL, MMA > 250 nmol/L).

### Assessment of hyperlipidemia

Hyperlipidemia was defined as total cholesterol ≥ 200 mg/dL; triglycerides ≥ 150 mg/dL; high-density lipoprotein ≤ 40 mg/dL in males and ≤ 50 mg/dL in females; or low-density lipoprotein ≥ 130 mg/dL (17). Additionally, participants who self-reported using cholesterol-lowering medications were also included.

### Assessment of covariates

The covariates included age, sex, race, education level, BMI, alcohol consumption, smoking status, and comorbidities such as stroke, coronary heart disease, diabetes, hypertension, and heart failure. Additionally, blood biochemical markers were considered, including blood urea nitrogen, albumin, uric acid, creatinine, fasting blood glucose, and glycated hemoglobin levels. Race was categorized as non-Hispanic Black, non-Hispanic White, Mexican American, and other races. Education levels were categorized as below high school, high school graduates, and above high school. Smokers were defined as individuals who have smoked a minimum of 100 cigarettes in their lifetime. Regarding alcohol consumption, it was classified into five categories based on previous research (18). Coronary heart disease was defined as the presence of either physician-diagnosed myocardial infarction or angina. Diabetes was defined as either a reported diagnosis of diabetes or current use of diabetes medication or insulin. Hypertension was defined as systolic blood pressure ≥ 140 mmHg, diastolic blood pressure ≥ 90 mmHg, or the use of antihypertensive therapy.

### Statistical analysis

The present investigation utilized MEC weights as the weighting variable in the analytical framework. Differences in characteristics were evaluated through weighted one-way ANOVA for continuous variables and weighted Chi-square tests for categorical variables. To guarantee a normal distribution for forthcoming analyses, serum MMA and vitamin B12 levels underwent log transformation (natural logarithm) (ln MMA, ln B12). Kaplan–Meier survival curves were delineated across the strata of ln B12 and ln MMA individually. Survey-weighted Cox proportional hazards regression models were employed to scrutinize the associations between all-cause mortality and ln MMA (categorized into tertiles), ln B12 (categorized into tertiles), and the combined serum vitamin B12 and MMA levels categorized into four distinct groups. Hazard ratios (HR) alongside 95% confidence intervals (CI) were utilized to encapsulate the findings. Model 1 did not incorporate any covariate adjustments; Model 2 was adjusted for age, sex, race, and educational attainment. Model 3 underwent further adjustment for BMI, alcohol use, smoking status, history of stroke, coronary heart disease, diabetes, hypertension, heart failure, blood urea nitrogen, albumin, uric acid, creatinine, blood glucose, and glycated hemoglobin. Multivariable-adjusted restricted cubic spline (RCS) analyses were conducted to probe potential nonlinear relationships between ln MMA, ln B12, and all-cause mortality. Statistical evaluations were executed using RStudio (Version 4.4.2), with *p*-values < 0.05 deemed statistically significant.

## Results

### Baseline characteristics

As detailed in Table 1, this study included a total of 6,617 hyperlipidemic patients aged 20 years or older. During a median follow-up of 6.8 years, 626 deaths (9.5%) were recorded. Comparisons between deceased and surviving participants revealed no significant differences in sex distribution or body mass index (BMI). However, the mean age was significantly higher in the deceased group (67 ± 14 years) than in the survival group (49 ± 16 years). The prevalence of comorbidities—including hypertension, heart failure, coronary heart disease, diabetes mellitus, and stroke—was also markedly elevated among deceased participants. Furthermore, a higher proportion of individuals in the deceased group had a history of smoking and former alcohol use. Critically, serum methylmalonic acid (MMA) levels were significantly higher in those who died (253 ± 200 nmol/L) compared to survivors (168 ± 117 nmol/L; *p* < 0.001). Although serum vitamin B12 levels appeared slightly elevated in the deceased group (747 ± 1,129 pg./mL vs. 608 ± 576 pg./mL), this difference was not statistically significant (*p* = 0.068). Consistent trends were observed when participants were stratified by tertiles of ln-transformed B12 and MMA (Supplementary Tables S1, S2). Combined groupings based on both MMA and vitamin B12 biomarkers are presented in Supplementary Table S3.

**Table 1:** Baseline characteristics of participants.

Characteristics	Overall	Survival	Death	*p* value
Number	6,617	5,991	626	
Age (years)	50 ± 16	49 ± 16	67 ± 14	<0.001
Sex (%)				0.500
Female	3,432 (52%)	3,133 (52%)	299 (50%)	
Male	3,185 (48%)	2,858 (48%)	327 (50%)	
Race (%)				<0.001
Non-Hispanic White	2,866 (70%)	2,500 (69%)	366 (79%)	
Non-Hispanic Black	1,335 (9.2%)	1,200 (9.2%)	135 (9.5%)	
Mexican American	790 (7.9%)	750 (8.3%)	40 (3.5%)	
Other races	1,626 (13%)	1,541 (14%)	85 (7.5%)	
Education level (%)				0.003
College or above	3,671 (63%)	3,401 (64%)	270 (52%)	
High school or equivalent	1,459 (21%)	1,299 (21%)	160 (25%)	
Less than high school	1,487 (16%)	1,291 (15%)	196 (22%)	
BMI (kg/m^2^)	30 ± 7	30 ± 7	30 ± 7	>0.900
SBP (mmHg)	123 ± 17	123 ± 16	133 ± 22	<0.001
DBP (mmHg)	71 ± 12	72 ± 12	67 ± 15	<0.001
Heart failure (%)	255 (3.2%)	149 (2.1%)	106 (17%)	<0.001
CHD (%)	538 (7.2%)	384 (5.9%)	154 (24%)	<0.001
Hypertension (%)	3,193 (44%)	2,708 (42%)	485 (75%)	<0.001
DM (%)	1,127 (13%)	915 (12%)	212 (31%)	<0.001
Stroke (%)	254 (3.0%)	171 (2.3%)	83 (11%)	<0.001
Smoking (%)	2,981 (46%)	2,613 (44%)	368 (60%)	<0.001
Alcohol drinking (%)				<0.001
None	1,005 (11%)	912 (11%)	93 (13%)	
Former	1,254 (16%)	1,013 (15%)	241 (34%)	
Mild	2,238 (37%)	2052 (37%)	186 (36%)	
Moderate	965 (17%)	924 (18%)	41 (7.9%)	
Heavy	1,155 (18%)	1,090 (19%)	65 (9.5%)	
Albumin (g/L)	43 ± 3	43 ± 3	42 ± 4	<0.001
ALT (U/L)	26 ± 23	26 ± 18	26 ± 55	0.800
Glucose (mg/dL)	104 ± 38	102 ± 36	118 ± 55	<0.001
BUN (mg/dL)	14 ± 6	13 ± 5	18 ± 10	<0.001
Cr (umol/L)	80 ± 37	78 ± 26	102 ± 94	<0.001
BIL (mg/dL)	0.67 ± 0.29	0.67 ± 0.29	0.69 ± 0.31	0.400
UA (umol/L)	139 ± 2	139 ± 2	139 ± 3	0.600
Glycohemoglobin (%)	5.73 ± 1.02	5.70 ± 0.98	6.16 ± 1.34	<0.001
Vitamin B12 (pg/mL)	619 ± 637	608 ± 576	747 ± 1,129	0.068
MMA (nmol/L)	174 ± 128	168 ± 117	253 ± 200	<0.001

All values were presented as mean ± SD, or counts (unweighted, proportion). BMI: Body Mass Index; SBP: Systolic Blood Pressure; DBP: Diastolic Blood Pressure; CHD: Coronary Heart Disease; DM: Diabetes Mellitus; ALT: Alanine Aminotransferase; BUN: Blood Urea Nitrogen; BIL: Bilirubin; UA: Uric Acid; MMA: Methylmalonic Acid.

### Independent association of MMA with all-cause mortality

**Figure 2:**
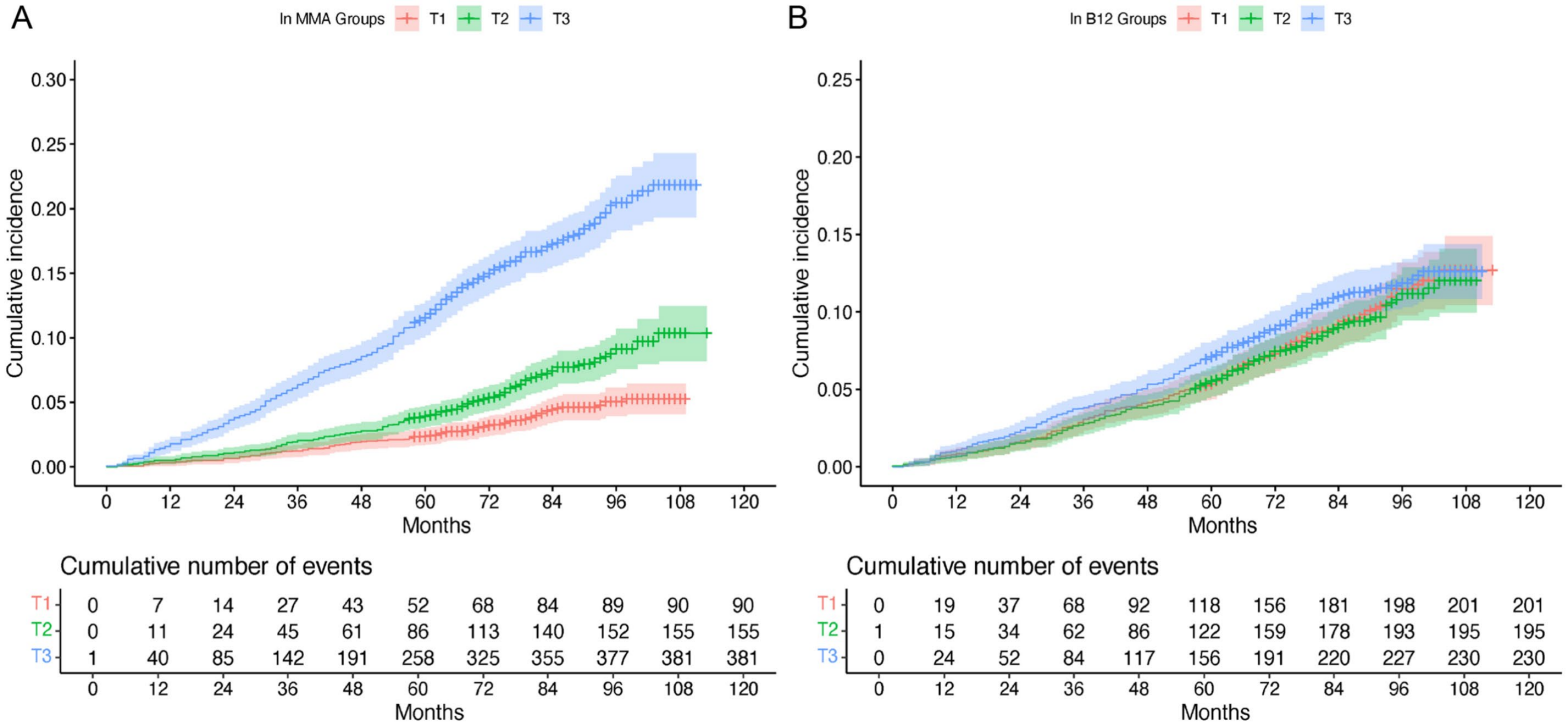
Cumulative all-cause mortality by ln MMA **(A)** and ln B12 **(B)** in adults with hyperlipidemia. MMA, Methylmalonic acid; B12, vitamin B12.

**Figure 3:**
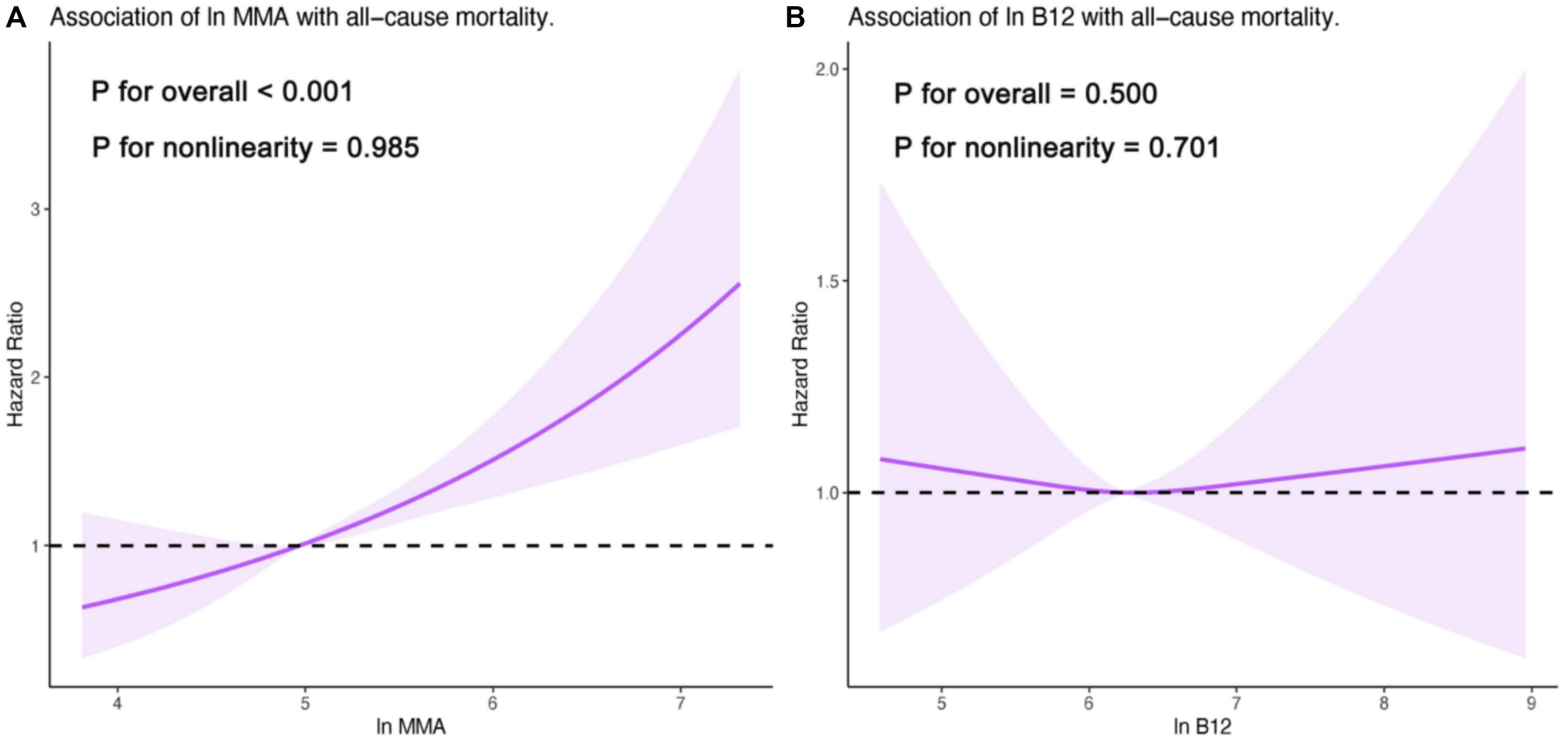
Association of ln MMA **(A)** and ln B12 **(B)** with all-cause mortality in adults with hyperlipidemia based on multivariable-adjusted restricted cubic spline models. Models were adjusted for age, sex, race, education level, BMI, alcohol drinking, smoking status, DM, CHD, HF, stroke, glucose, serum creatinine, uric acid, BUN, HbA1c, and albumin. MMA, methylmalonic acid; B12, vitamin B12.

Cox proportional hazards and Kaplan–Meier analyses consistently indicated a strong positive association between ln MMA and all-cause mortality (Table 2; Figure 2A). After multivariable adjustment, each one-unit increase in ln MMA was associated with a 55% elevated risk of mortality (HR = 1.55; 95% CI: 1.30–1.84; *p* < 0.001). Tertile-based comparisons revealed a graded increase in mortality risk with increasing ln MMA levels. Participants in the highest tertile had a significantly increased risk of death compared to those in the lowest tertile (adjusted HR = 1.58; 95% CI: 1.10–2.26; *p* < 0.001), with a significant trend across tertiles (P-trend < 0.001). Restricted cubic spline (RCS) analysis confirmed a linear dose–response relationship between ln MMA and mortality (P for nonlinearity = 0.985; Figure 3A).

**Table 2:** Association of methylmalonic acid and vitamin B12 with all-cause mortality among adults with hyperlipidemia.

Variable	Model 1		Model 2		Model 3	
	HR (95% CI)	*P* value	HR (95% CI)	*P* value	HR (95% CI)	*P* value
Methylmalonic acid						
ln MMA	3.19 (2.77, 3.67)	<0.001	1.88 (1.59, 2.23)	<0.001	1.55 (1.30, 1.84)	<0.001
T1	Ref.		Ref.		Ref.	
T2	1.55 (1.04, 2.31)	0.033	1.04 (0.70, 1.55)	0.800	1.01 (0.67, 1.51)	0.900
T3	4.48 (3.25, 6.18)	<0.001	1.90 (1.33, 2.73)	<0.001	1.58 (1.10, 2.26)	0.014
*P* for trend		<0.001		<0.001		0.002
Vitamin B12						
ln B12	1.32 (1.05, 1.65)	0.017	1.05 (0.85, 1.31)	0.600	1.07 (0.87, 1.32)	0.500
T1	Ref.		Ref.		Ref.	
T2	1.01 (0.80, 1.27)	0.900	0.95 (0.73, 1.26)	0.700	1.07 (0.85, 1.34)	0.600
T3	1.37 (1.03, 1.82)	0.033	1.08 (0.78, 1.50)	0.600	1.10 (0.82, 1.48)	0.500
*P* for trend		0.037		0.600		0.500

Model 1: adjusted for none.

Model 2: adjusted for age, sex, race, education level.

Model 3: adjusted for age, sex, race, education level, BMI, alcohol drinking, smoking status, DM, CHD, HF, stroke, glucose, serum creatinine, uric acid, BUN, HbA1c, and albumin.

BMI, body mass index; CHD, coronary heart disease; DM, diabetes mellitus; HF, heart failure; BUN, blood urea nitrogen; HbA1c, glycated hemoglobin.

### Independent association of vitamin B12 with all-cause mortality

In contrast, no significant association was observed between serum vitamin B12 (ln B12) and all-cause mortality in this hyperlipidemic cohort. The fully adjusted hazard ratio per unit increase in ln B12 was 1.07 (95% CI: 0.87–1.32; *p* = 0.500). Similarly, when comparing the highest to the lowest tertile of ln B12, the multivariable-adjusted HR was 1.10 (95% CI: 0.82–1.48; *p* = 0.500). Kaplan–Meier curves further supported the absence of a significant survival difference across ln B12 strata (Figure 2B). RCS analysis indicated no evidence of a nonlinear association (P for nonlinearity = 0.701; Figure 3B).

### Combined effects of serum MMA and vitamin B12 on all-cause mortality

**Figure 4:**
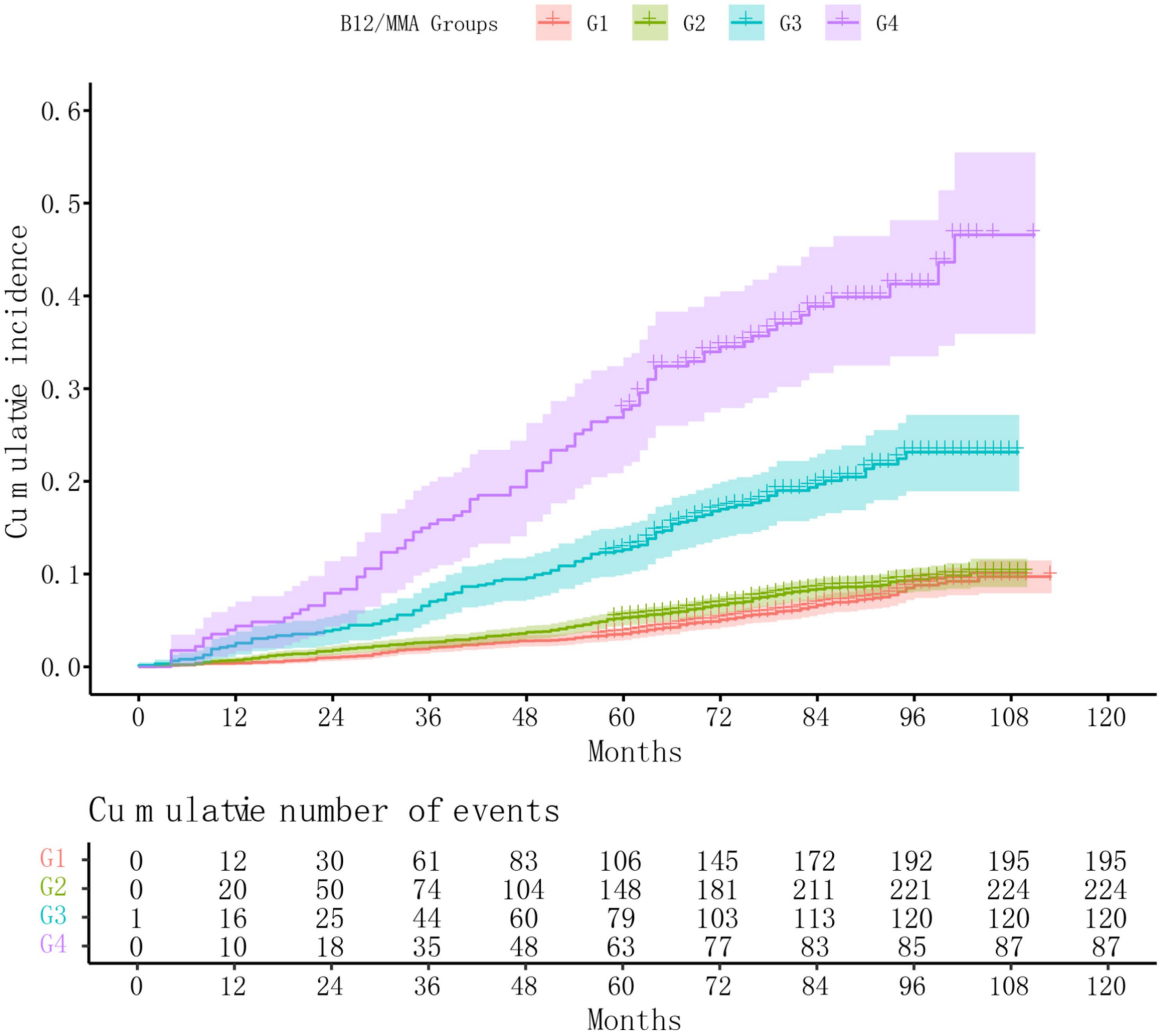
Kaplan–Meier survival curves by combined B12 and MMA status. participants were categorized into four groups based on cutoff values of serum MMA and B12: G1 (B12_low_MMA_low_, vitamin B12 < 400 pg./mL and MMA < 250 nmol/L), G2 (B12_high_MMA_low_, vitamin B12 > 400 pg./mL and MMA < 250 nmol/L), G3 (B12_low_MMA_high_, vitamin B12 < 400 pg./mL and MMA > 250 nmol/L), and G4 (B12_high_MMA_high_, vitamin B12 > 400 pg./mL and MMA > 250 nmol/L). MMA, methylmalonic acid; B12, vitamin B12.

Stratified analyses based on combined MMA and vitamin B12 levels yielded notable findings (Table 3). After full adjustment, individuals in the B12_low_MMA_high_ group showed a significantly elevated mortality risk compared to the B12_low_MMA_low_ reference group (HR = 1.41; 95% CI: 1.03–1.93; *p* = 0.03). Most strikingly, participants with high levels of both MMA and B12 (B12_high_MMA_high_) faced the highest risk of death (HR = 2.40; 95% CI: 1.70–3.41; *p* < 0.001). No significant difference was observed between the B12_high_MMA_low_ and B12_low_MMA_low_ groups (*p* = 0.4). These results were corroborated by Kaplan–Meier survival analyses (Figure 4).

**Table 3:** Joint association of methylmalonic acid and vitamin B12 with all-cause mortality among adults with hyperlipidemia.

Variable	Model 1		Model 2		Model 3	
	HR (95% CI)	*P* value	HR (95% CI)	*P* value	HR (95% CI)	*P* value
B12/MMA groups						
B12_low_MMA_low_	Ref.		Ref.		Ref.	
B12_high_MMA_low_	1.42 (1.03, 1.97)	0.033	1.11 (0.79, 1.56)	0.500	1.15 (0.81, 1.61)	0.400
B12_low_MMA_high_	3.04 (2.28, 4.05)	<0.001	1.61 (1.25, 2.09)	<0.001	1.41 (1.03, 1.93)	0.030
B12_high_MMA_high_	8.64 (5.98, 12.5)	<0.001	3.20 (2.21, 4.63)	<0.001	2.40 (1.70, 3.41)	<0.001
*P* for trend		<0.001		<0.001		<0.001

Model 1: adjusted for none.

Model 2: adjusted for age, sex, race, education level.

Model 3: adjusted for age, sex, race, education level, BMI, alcohol drinking, smoking status, DM, CHD, HF, stroke, glucose, serum creatinine, uric acid, BUN, HbA1c, and albumin.

BMI, body mass index; CHD, coronary heart disease; DM, diabetes mellitus; HF, heart failure; BUN, blood urea nitrogen; HbA1c, glycated hemoglobin.

## Discussion

This large-scale cohort study provides robust evidence on the relationship between serum methylmalonic acid (MMA), vitamin B12, and all-cause mortality among individuals with hyperlipidemia. After comprehensive adjustment for potential confounders, elevated ln-transformed MMA levels were significantly associated with an increased risk of all-cause mortality, whereas no independent association was observed for serum vitamin B12 levels alone. Notably, the combined analysis revealed that individuals with high MMA levels—particularly those with concurrently high vitamin B12—faced the greatest mortality risk, suggesting the presence of functional vitamin B12 deficiency. These findings underscore the clinical relevance of MMA as a prognostic biomarker and highlight the importance of moving beyond serum B12 measurement alone in risk stratification.

MMA plays a multifaceted role in lipid metabolism. Experimental studies in mice indicate that MMA suppresses hepatic cholesterol synthesis and upregulates LDL receptor activity, thereby lowering plasma cholesterol (14). Long-term MMA administration also reduced plasma triglycerides and brain myelin content (19). Despite these mechanistic insights, the clinical relevance of MMA in human hyperlipidemia remains poorly understood. A small cross-sectional study indicated that vitamin B12 deficiency is frequent among hyperlipidemic patients, hinting at a possible metabolic connection (20). Although MMA may exert beneficial effects on lipid profiles in animal models, its accumulation, as a metabolic byproduct, may have adverse implications in humans. Given interspecies differences in lipoprotein metabolism, further investigation is warranted to elucidate the role of MMA in the prognosis of hyperlipidemic patients.

Elevated MMA levels are associated with aging and multiple chronic conditions (21). In older adults, increased MMA may reflect organ dysfunction and progression of age-related diseases (15), though the direction of causality remains unclear. However, a causal relationship between aging and elevated MMA levels remains unclear. MMA has been extensively implicated in neurodegenerative disorders and cognitive decline (16, 17), with significant links to mortality from Alzheimer’s and Parkinson’s diseases (18). It is also correlated with liver fibrosis severity, a hallmark of non-alcoholic fatty liver disease (22, 23), and serves as an independent risk factor for mortality in NAFLD patients (9). Moreover, elevated MMA predicts adverse cardiovascular outcomes; it is heightened in heart failure patients (24) and associated with acute myocardial infarction and all-cause mortality in coronary artery disease (10). MMA has also been linked to increased mortality in cancer, chronic kidney disease, and diabetes (25–27). Importantly, our analysis specifically demonstrates that elevated MMA and functional B12 deficiency are robust, independent predictors of all-cause mortality in the context of hyperlipidemia. To our knowledge, this is the first study to establish MMA as a promising biomarker for mortality risk stratification specifically in a large hyperlipidemic cohort.

The mechanisms through which elevated MMA increases mortality risk in hyperlipidemia may involve mitochondrial dysfunction, oxidative stress, and inflammation. First, MMA inhibits mitochondrial respiratory chain complexes and disrupts mitophagy, propagating mitochondrial damage (28, 29). Second, it amplifies oxidative stress by activating cancer-associated fibroblasts and immune cells to produce reactive oxygen species and pro-inflammatory cytokines (30, 31). Third, MMA-mediated activation of the succinate receptor may promote inflammatory pathways (32, 33), potentially exacerbating metabolic and cardiovascular pathologies. Although elevated MMA is common in aging and malignancy (15, 21, 34), its causal role remains uncertain. Furthermore, this pathophysiological cascade may be particularly deleterious in the context of hyperlipidemia. A recent analysis of the NHANES cohort indicated that MMA mediates the association between oxidative stress and cardiovascular risk in individuals with dyslipidemia, accounting for a significant proportion (14.9%) of this risk. This suggests that elevated MMA may act as a critical metabolic link, amplifying oxidative damage and inflammatory pathways to accelerate cardiovascular mortality in this susceptible patient population (35). Further research is needed to clarify whether MMA is a mediator or marker of pathological processes and to explore its therapeutic relevance.

The absence of an association between serum vitamin B12 and mortality aligns with prior conflicting reports (36–38). Serum B12 levels may be influenced by binding proteins and immunoglobulins and do not reliably reflect intracellular status or functional adequacy (39). Current guidelines recommend incorporating metabolic markers such as MMA, which offers superior sensitivity and specificity for detecting functional vitamin B12 deficiency (6). Our finding that the B12_high_MMA_high_ subgroup had the highest mortality risk underscores the concept of “impaired vitamin B12 sensitivity,” where cellular uptake, transport, or utilization of B12 is compromised despite normal or high serum levels (40). This phenomenon has been documented in diabetes, coronary artery disease, and NAFLD (9–11) and is linked accelerated biological aging (12). Whether B12 supplementation benefits such patients remains unclear and merits further investigation. Our finding that serum B12 concentration alone did not show an independent association with all-cause mortality, while functional B12 deficiency conferred the highest risk, provides crucial mechanistic and clinical insight. This discrepancy likely arises for several reasons. First, it underscores a key limitation of relying solely on serum B12 as a biomarker of functional vitamin B12 status at the tissue and cellular level. Serum B12 levels may remain within the normal range despite intracellular deficiency or metabolic dysfunction, a state revealed by elevated MMA. Second, prior studies reporting a link between low B12 and mortality often included populations with a higher prevalence of overt nutritional deficiency. Our study focused specifically on hyperlipidemic patients within a generally nourished population (NHANES), where severe B12 deficiency is less common. In this context, MMA, reflecting the functional metabolic consequence of B12-dependent pathways, may be a more sensitive and specific marker of biologically relevant dysfunction. This is powerfully supported by our result that the combination of high MMA with normal/high B12 identified the subgroup at greatest risk. Therefore, our data suggest that in populations similar to our cohort, assessment of functional B12 status using MMA is prognostically superior to measurement of serum B12 alone.

A key strength of this study is its use of a large, nationally representative cohort with long-term follow-up, extensive covariate adjustment, and novel evaluation of vitamin B12 sensitivity using combined MMA and B12 levels. Notably, this study provides the first large-scale, prospective evidence specifically examining the prognostic value of serum MMA within a hyperlipidemic population, thereby addressing a significant gap in the literature regarding novel biomarkers for residual risk stratification in this high-risk group. However, several limitations should be acknowledged. The observational design precludes causal inference. The sample was derived from a U. S. population, which may limit generalizability to other ethnic or geographic groups. Although we adjusted for numerous confounders, residual confounding due to unmeasured factors (e.g., dietary habits, genetic background) cannot be excluded.

## Conclusion

This prospective study demonstrates that elevated MMA levels are strongly associated with increased all-cause mortality in individuals with hyperlipidemia, particularly in the context of impaired vitamin B12 sensitivity. MMA may serve as a novel biomarker for mortality risk in hyperlipidemia population. These findings advocate for the incorporation of MMA into clinical risk assessment tools and highlight the need to better understand and address functional B12 deficiency in high-risk populations.

## Data Availability

The original contributions presented in the study are included in the article/Supplementary material, further inquiries can be directed to the corresponding authors.
