# Supplementary material for: Elevated methylmalonic acid, but not vitamin B12, predicts all-cause mortality in hyperlipidemic adults: a prospective cohort study
Source: Front Nutr. 2026 Jan 15;13:1742540. doi: 10.3389/fnut.2026.1742540 (PMC12852000; doi:10.3389/fnut.2026.1742540)
Supplement: Supplementary file 1 [file Table_1.DOCX]

Table S1. Baseline characteristics of patients grouped by ln MMA.

| Characteristics | T1: [3.28, 4.80] | T2: (4.80, 5.15] | T3: (5.15, 8.62] | *P* value |
| --- | --- | --- | --- | --- |
| Number | 2195 | 2190 | 2232 |  |
| Age (years) | 44 ± 15 | 49 ± 16 | 56 ± 16 | <0.001 |
| Sex (%) |  |  |  | 0.066 |
| Female | 1,218 (55%) | 1,083 (49%) | 1,131 (52%) |  |
| Male | 977 (45%) | 1,107 (51%) | 1,101 (48%) |  |
| Race (%) |  |  |  | <0.001 |
| Non-Hispanic White | 605 (53%) | 1,008 (74%) | 1,253 (80%) |  |
| Non-Hispanic Black | 580 (15%) | 437 (8.0%) | 318 (5.7%) |  |
| Mexican American | 383 (15%) | 225 (6.0%) | 182 (4.0%) |  |
| Other Races | 627 (17%) | 520 (12%) | 479 (11%) |  |
| Education level (%) |  |  |  | 0.083 |
| College or above | 1,257 (65%) | 1,235 (64%) | 1,179 (60%) |  |
| High school or equivalent | 465 (20%) | 509 (22%) | 485 (22%) |  |
| Less than high school | 473 (16%) | 446 (14%) | 568 (17%) |  |
| BMI (kg/m^2^) | 30 ± 7 | 30 ± 6 | 30 ± 7 | 0.3 |
| SBP (mmHg) | 121 ± 15 | 123 ± 15 | 126 ± 19 | <0.001 |
| DBP (mmHg) | 72 ± 11 | 72 ± 11 | 70 ± 13 | 0.007 |
| Heart failure (%) | 31 (1.2%) | 65 (2.5%) | 159 (5.8%) | <0.001 |
| CHD (%) | 90 (3.8%) | 158 (6.4%) | 290 (11%) | <0.001 |
| Hypetension (%) | 852 (36%) | 1,043 (43%) | 1,298 (53%) | <0.001 |
| DM (%) | 338 (12%) | 327 (12%) | 462 (16%) | 0.002 |
| Stroke (%) | 41 (1.6%) | 64 (2.0%) | 149 (5.2%) | <0.001 |
| Smoking (%) | 867 (40%) | 1,026 (47%) | 1,088 (49%) | <0.001 |
| Alcohol drinking (%) |  |  |  | <0.001 |
| None | 335 (12%) | 300 (9.7%) | 370 (13%) |  |
| Former | 336 (14%) | 391 (16%) | 527 (19%) |  |
| Mild | 693 (34%) | 763 (37%) | 782 (40%) |  |
| Moderate | 355 (18%) | 355 (19%) | 255 (14%) |  |
| Heavy | 476 (23%) | 381 (19%) | 298 (14%) |  |
| Albumin (g/L) | 43 ± 4 | 43 ± 3 | 43 ± 3 | <0.001 |
| ALT (U/L) | 27 ± 19 | 26 ± 18 | 25 ± 29 | 0.002 |
| Glucose (mg/dL) | 101 ± 35 | 103 ± 37 | 106 ± 41 | <0.001 |
| BUN (mg/dL) | 12 ± 4 | 13 ± 4 | 16 ± 7 | <0.001 |
| Cr (umol/L) | 72 ± 17 | 78 ± 17 | 89 ± 57 | <0.001 |
| BIL (mg/dL) | 0.67 ± 0.30 | 0.68 ± 0.29 | 0.66 ± 0.29 | 0.12 |
| UA (umol/L) | 139 ± 2 | 139 ± 2 | 139 ± 3 | 0.09 |
| Glycohemoglobin (%) | 5.68 ± 1.01 | 5.70 ± 0.97 | 5.81 ± 1.07 | <0.001 |
| Vitamin B12 (pg/mL) | 726 ± 885 | 626 ± 521 | 519 ± 451 | <0.001 |
| MMA (nmol/L) | 97 ± 17 | 145 ± 15 | 271 ± 176 | <0.001 |

All values were presented as mean ± SD, or counts (unweighted, proportion). BMI: Body Mass Index; SBP: Systolic Blood Pressure; DBP: Diastolic Blood Pressure; CHD: Coronary Heart Disease; DM: Diabetes Mellitus; ALT: Alanine Aminotransferase; BUN: Blood Urea Nitrogen; BIL: Bilirubin; UA: Uric Acid; MMA: Methylmalonic Acid.

Table S2. Baseline characteristics of patients grouped by ln B12.

| Characteristics | T1: [2.89, 6.05] | T2: (6.05, 6.45] | T3: (6.45, 10.20] | *P* value |
| --- | --- | --- | --- | --- |
| Number | 2211 | 2205 | 2201 |  |
| Age (years) | 49 ± 16 | 49 ± 16 | 53 ± 17 | <0.001 |
| Sex (%) |  |  |  | <0.001 |
| Female | 1,109 (53%) | 1,043 (46%) | 1,280 (57%) |  |
| Male | 1,102 (47%) | 1,162 (54%) | 921 (43%) |  |
| Race (%) |  |  |  | <0.001 |
| Non-Hispanic White | 1,035 (72%) | 975 (70%) | 856 (67%) |  |
| Non-Hispanic Black | 386 (7.7%) | 431 (9.0%) | 518 (11%) |  |
| Mexican American | 251 (7.1%) | 292 (9.0%) | 247 (7.6%) |  |
| Other Races | 539 (13%) | 507 (12%) | 580 (14%) |  |
| Education level (%) |  |  |  | 0.5 |
| College or above | 1,238 (64%) | 1,182 (61%) | 1,251 (64%) |  |
| High school or equivalent | 475 (20%) | 503 (23%) | 481 (21%) |  |
| Less than high school | 498 (16%) | 520 (16%) | 469 (15%) |  |
| BMI (kg/m^2^) | 31 ± 7 | 30 ± 7 | 29 ± 7 | <0.001 |
| SBP (mmHg) | 124 ± 17 | 123 ± 16 | 124 ± 18 | 0.3 |
| DBP (mmHg) | 72 ± 12 | 72 ± 12 | 70 ± 12 | 0.035 |
| Heart failure (%) | 86 (3.4%) | 71 (2.5%) | 98 (3.8%) | 0.11 |
| CHD (%) | 178 (7.0%) | 182 (7.5%) | 178 (7.1%) | 0.8 |
| Hypetension (%) | 1,044 (45%) | 1,019 (42%) | 1,130 (46%) | 0.3 |
| DM (%) | 361 (14%) | 337 (11%) | 429 (16%) | 0.002 |
| Stroke (%) | 88 (3.0%) | 71 (2.4%) | 95 (3.7%) | 0.2 |
| Smoking (%) | 1,022 (46%) | 1,024 (47%) | 935 (43%) | 0.13 |
| Alcohol drinking (%) |  |  |  | <0.001 |
| None | 342 (12%) | 303 (9.8%) | 360 (12%) |  |
| Former | 417 (16%) | 403 (15%) | 434 (18%) |  |
| Mild | 700 (35%) | 754 (38%) | 784 (39%) |  |
| Moderate | 346 (19%) | 332 (17%) | 287 (15%) |  |
| Heavy | 406 (19%) | 413 (20%) | 336 (16%) |  |
| Albumin (g/L) | 42 ± 3 | 43 ± 3 | 43 ± 3 | <0.001 |
| ALT (U/L) | 24 ± 14 | 26 ± 17 | 28 ± 33 | <0.001 |
| Glucose (mg/dL) | 104 ± 38 | 103 ± 34 | 104 ± 41 | 0.7 |
| BUN (mg/dL) | 13 ± 5 | 13 ± 5 | 14 ± 7 | <0.001 |
| Cr (umol/L) | 79 ± 40 | 79 ± 22 | 82 ± 46 | 0.2 |
| BIL (mg/dL) | 0.66 ± 0.29 | 0.67 ± 0.31 | 0.68 ± 0.28 | 0.12 |
| UA (umol/L) | 139 ± 2 | 139 ± 2 | 139 ± 2 | 0.5 |
| Glycohemoglobin (%) | 5.72 ± 0.99 | 5.68 ± 0.95 | 5.80 ± 1.11 | 0.015 |
| vitamin B12 (pg/mL) | 325 ± 70 | 520 ± 60 | 1,053 ± 999 | <0.001 |
| MMA (nmol/L) | 213 ± 181 | 157 ± 76 | 149 ± 82 | <0.001 |

All values were presented as mean ± SD, or counts (unweighted, proportion). BMI: Body Mass Index; SBP: Systolic Blood Pressure; DBP: Diastolic Blood Pressure; CHD: Coronary Heart Disease; DM: Diabetes Mellitus; ALT: Alanine Aminotransferase; BUN: Blood Urea Nitrogen; BIL: Bilirubin; UA: Uric Acid; MMA: Methylmalonic Acid; B12: vitamin B12.

Table S3. Baseline characteristics of patients stratified by serum MMA and vitamin B12 levels.

| Characteristics | B12_low_MMA_low_ | B12_high_MMA_low_ | B12_low_MMA_high_ | B12_high_MMA_high_ | P value |
| --- | --- | --- | --- | --- | --- |
| Number | 2976 | 2789 | 625 | 227 |  |
| Age (years) | 47 ± 16 | 51 ± 17 | 57 ± 16 | 64 ± 15 | <0.001 |
| Sex (%) |  |  |  |  | 0.4 |
| Female | 1,458 (50%) | 1,538 (53%) | 313 (50%) | 123 (57%) |  |
| Male | 1,518 (50%) | 1,251 (47%) | 312 (50%) | 104 (43%) |  |
| Race (%) |  |  |  |  | <0.001 |
| Non-Hispanic White | 1,312 (70%) | 1,082 (67%) | 335 (77%) | 137 (83%) |  |
| Non-Hispanic Black | 581 (8.7%) | 645 (11%) | 73 (4.9%) | 36 (6.4%) |  |
| Mexican American | 380 (8.6%) | 335 (8.2%) | 61 (4.6%) | 14 (2.9%) |  |
| Other Races | 703 (13%) | 727 (14%) | 156 (14%) | 40 (7.8%) |  |
| Education level (%) |  |  |  |  | 0.081 |
| College or above | 1,660 (64%) | 1,580 (63%) | 313 (58%) | 118 (56%) |  |
| High school or equivalent | 670 (21%) | 610 (21%) | 131 (21%) | 48 (27%) |  |
| Less than high school | 646 (14%) | 599 (15%) | 181 (21%) | 61 (18%) |  |
| BMI (kg/m2) | 31 ± 7 | 29 ± 6 | 30 ± 7 | 29 ± 7 | <0.001 |
| SBP (mmHg) | 123 ± 16 | 123 ± 16 | 127 ± 21 | 128 ± 24 | 0.002 |
| DBP (mmHg) | 72 ± 12 | 71 ± 12 | 70 ± 15 | 67 ± 13 | <0.001 |
| Heart failure (%) | 81 (2.4%) | 93 (2.8%) | 46 (6.2%) | 35 (13%) | <0.001 |
| CHD (%) | 213 (6.5%) | 205 (6.7%) | 69 (9.0%) | 51 (20%) | <0.001 |
| Hypetension (%) | 1,325 (42%) | 1,318 (43%) | 373 (55%) | 177 (71%) | <0.001 |
| DM (%) | 432 (11%) | 485 (13%) | 139 (19%) | 71 (27%) | <0.001 |
| Stroke (%) | 86 (2.0%) | 90 (2.8%) | 47 (6.6%) | 31 (11%) | <0.001 |
| Smoking (%) | 1,372 (46%) | 1,197 (45%) | 293 (47%) | 119 (49%) | 0.6 |
| Alcohol drinking (%) |  |  |  |  | <0.001 |
| None | 418 (10%) | 426 (11%) | 118 (15%) | 43 (15%) |  |
| Former | 510 (14%) | 522 (17%) | 159 (20%) | 63 (21%) |  |
| Mild | 960 (35%) | 988 (39%) | 203 (37%) | 87 (45%) |  |
| Moderate | 493 (19%) | 394 (16%) | 65 (14%) | 13 (7.9%) |  |
| Heavy | 595 (21%) | 459 (17%) | 80 (14%) | 21 (9.7%) |  |
| Albumin (g/L) | 43 ± 3 | 43 ± 3 | 42 ± 3 | 42 ± 3 | <0.001 |
| ALT (U/L) | 25 ± 16 | 28 ± 30 | 22 ± 10 | 25 ± 17 | <0.001 |
| Glucose (mg/dL) | 103 ± 35 | 103 ± 39 | 110 ± 42 | 113 ± 42 | <0.001 |
| BUN (mg/dL) | 13 ± 4 | 14 ± 5 | 15 ± 8 | 22 ± 11 | <0.001 |
| Cr (umol/L) | 76 ± 18 | 78 ± 20 | 91 ± 73 | 128 ± 125 | <0.001 |
| BIL (mg/dL) | 0.67 ± 0.29 | 0.68 ± 0.30 | 0.65 ± 0.28 | 0.68 ± 0.23 | 0.3 |
| UA (umol/L) | 139 ± 2 | 139 ± 2 | 139 ± 2 | 139 ± 3 | 0.5 |
| Glycohemoglobin (%) | 5.67 ± 0.94 | 5.75 ± 1.06 | 5.91 ± 1.15 | 5.97 ± 1.05 | <0.001 |
| Vitamin B12 (pg/mL) | 396 ± 90 | 921 ± 872 | 332 ± 106 | 893 ± 839 | <0.001 |
| MMA (nmol/L) | 149 ± 44 | 135 ± 42 | 418 ± 273 | 344 ± 145 | <0.001 |

All values were presented as mean ± SD, or counts (unweighted, proportion). BMI: Body Mass Index; SBP: Systolic Blood Pressure; DBP: Diastolic Blood Pressure; CHD: Coronary Heart Disease; DM: Diabetes Mellitus; ALT: Alanine Aminotransferase; BUN: Blood Urea Nitrogen; BIL: Bilirubin; UA: Uric Acid; MMA: Methylmalonic Acid.
